# *In vitro* Anticancer Activity of the Polar Fraction From the *Lophocereus schottii* Ethanolic Extract

**DOI:** 10.3389/fphar.2022.820381

**Published:** 2022-04-04

**Authors:** Arturo Orozco-Barocio, Blanca Susana Robles-Rodríguez, María del Rayo Camacho-Corona, Luis Fernando Méndez-López, Marisol Godínez-Rubí, Jorge Peregrina-Sandoval, Gildardo Rivera, Argelia E. Rojas Mayorquín, Daniel Ortuno-Sahagun

**Affiliations:** ^1^ Laboratorio de Inmunobiología, Departamento de Biología Celular y Molecular, Universidad de Guadalajara, Centro Universitario de Ciencias Biológicas y Agropecuarias, Zapopan, Mexico; ^2^ Facultad de Ciencias Químicas, Universidad Autónoma de Nuevo León, San Nicolás de los Garza, Mexico; ^3^ Centro de Investigación en Nutrición y Salud Publica, Facultad de Salud Pública y Nutrición, Universidad Autónoma de Nuevo León, San Nicolás de los Garza, Mexico; ^4^ Laboratorio de Patología Diagnóstica e Inmunohistoquímica, Departamento de Microbiología y Patología, CUCS, Universidad de Guadalajara, Guadalajara, Mexico; ^5^ Laboratorio de Biotecnología Farmacéutica, Centro de Biotecnología Genómica- Instituto Politécnico Nacional, Reynosa, Mexico; ^6^ Departamento de Ciencias Ambientales, Universidad de Guadalajara, Centro Universitario de Ciencias Biológicas y Agropecuarias, Zapopan, Mexico; ^7^ Laboratorio de Neuroinmunobiología Molecular, Departamento de Biología Molecular y Genómica, Instituto de Investigación en Ciencias Biomédicas (IICB), Universidad de Guadalajara, Centro Universitario de Ciencias de la Salud, Guadalajara, Mexico

**Keywords:** L5178Y lymphoma, *Lophocereus schottii*, cytotoxicity, cell viability, traditional medicine, anticancerogenic compounds

## Abstract

Cancer is an increasingly common disease and is considered one of the main causes of death in the world. *Lophocereus schottii* (*L. schottii*) is a cactus used in Mexico in traditional medicine for cancer treatment. This study aimed to determine the effect of the ethanolic extract and the polar and nonpolar fractions of *L. schottii* in murine L5178Y lymphoma cells *in vitro*, analyzing their effect on the proliferative activity of splenocytes, and establishing the effective concentration 50 (EC_50_) of the polar fraction. In addition, the secondary metabolites present in the extracts were determined by ultra-performance liquid chromatography-mass spectrometry (UPLC-MS). The study establishes that the three extracts of *L. schottii* have a cytotoxic effect on L5178Y cells and on the splenocytes stimulated with ConA. Additionally, the polar fraction has a significantly greater effect being three times more effective than cyclophosphamide on inhibiting the viability of L5178Y cells. Secondary metabolites present are mainly flavonoids and alkaloids, but there are also some terpenoids and sterols. Ultimately, polar fraction can be considered an anticancer substance, since its EC_50_ of 15 μg/mL is within the parameters established by the National Cancer Institute.

## Introduction

One recent area of interest for cancer treatment has been the study of plant-derived anticancerogenic (Ijaz et al., 2018) and immunomodulator compounds (Ortuño-Sahagún et al., 2017; Ortuño-Sahagún et al., 2019). Since the clinical use of chemotherapeutic agents has not met expectations in the treatment of many cancer types, there is a constant demand for the development of new treatments and new drugs (Solowey et al., 2014; Majolo et al., 2019).

Natural products derived from plants, or phytochemicals and their derivatives, have been used traditionally in natural medicine for centuries (Rizeq et al., 2020), demonstrating high potential for the development of new, natural anticancer drugs (Majolo et al., 2019; Choudhari et al., 2020). In fact, 42% of the total number of molecules approved for cancer treatment between 1981 and 2019 were either natural products or derived from them. It is significant that over the same period, 114 of the 247 anticancer molecules released (46%) were obtained from natural products and their derivatives (Newman and Cragg, 2020).

Metastasis, tumor growth, and multidrug resistance are the major causes of death in cancer patients and despite the diversity of chemotherapeutic drugs available, adverse effects, regressions, and failures in therapeutic response continue to persist. A single plant may contain a large quantity of secondary metabolites with varied therapeutic features, acting both individually and in combination or in synergy (Majolo et al., 2019; Costea et al., 2020). In fact, it has been proposed that the anti-cancer effect of some phytochemicals resides in their combined activity (Linnewiel-Hermoni et al., 2015).

In biological terms, Mexico is considered one of the megadiverse countries in the world. There are estimated to be some 23,400 vascular plants in the country, of which 3000 have medicinal effects (Alonso-Castro et al., 2017). Between 30 and 70% of cancer patients in Mexico use herbal extracts as an alternative therapy for many types of cancer (Alonso-Castro et al., 2011). One of the cactaceae species employed is *Lophocereus schottii (Engelm) Britton* & *Rose*. This cactus has traditionally been used for the treatment of cancer and other diseases including diabetes, ulcers, sores, stomach disorders, and tuberculosis (Argueta et al., 1994; Bravo Hollis and Scheinvar, 1995). Despite this, its precise chemical composition is still unknown.

In previous studies performed in our laboratory using inoculated mice with murine L5178Y lymphoma in the gastrocnemius muscle, we demonstrated an increased survival time and a decreased tumor mass in mice treated orally, intratumorally, and intramuscularly with the ethanolic extract of *L. schottii* stem (Orozco-Barocio et al., 2013). In the present study we focused on determining the effect of the ethanolic extract and the polar and nonpolar fractions of *L. schottii* in murine L5178Y lymphoma cells *in vitro*. Additionally, we studied their effect on the proliferative activity of splenocytes from healthy mice stimulated *in vitro* with mitogen concanavalin A (ConA). We established the effective concentration 50 (EC_50_) of the polar fraction in L5178Y murine lymphoma cells. Furthermore, we identified, by ultra-performance liquid chromatography-mass spectrometry (UPLC-MS), the main secondary metabolites present in the ethanolic extract and the different fractions.

## Materials and Methods

### Plant Material and Preparation of the Extract and Fractions

A piece of *Lophocereus schottii* (Engelm.) Britton & Rose stem was acquired in the “San Juan de Dios” market in the municipality of Guadalajara, Jalisco, Mexico and identified with the specimen stored in the Botanical Institute of the University Center for Biological and Agricultural Sciences (CUCBA) of the University of Guadalajara, with voucher number IBUG-189957. The specimen was collected fresh in August. Geographic coordinates: 20°40′32.1¨ N, 103°20′20.6¨ W. The piece was 50 cm long and 8 cm in diameter, weighing 1.5 kg. The plant material was sliced in pieces and dried in darkness at room temperature over 72 h; after that, it was immersed in ethanol 1:10 W/V. The ethanolic extract of *L. schottii* was obtained by extracting small, dry pieces of the stem in absolute ethanol (1:10 w/v) under stirring for 48 h at room temperature. The polar and nonpolar extracts were obtained by fractionation with hexane, at a 1:2 ratio, and both fractions were divided with a separatory funnel. The two fractions and the ethanolic extract were concentrated under low pressure in a rotary evaporator (Mod. RE47, Yamato Scientific Co., LTD, Tokyo, Japan), to a minimum volume (20 mL) in dark refrigerated bottles. The yield obtained (w/v) was: 0.72% for the ethanolic extract, 0.65% for the polar fraction, and 0.0526% for the nonpolar fraction.

**FIGURE 1:**
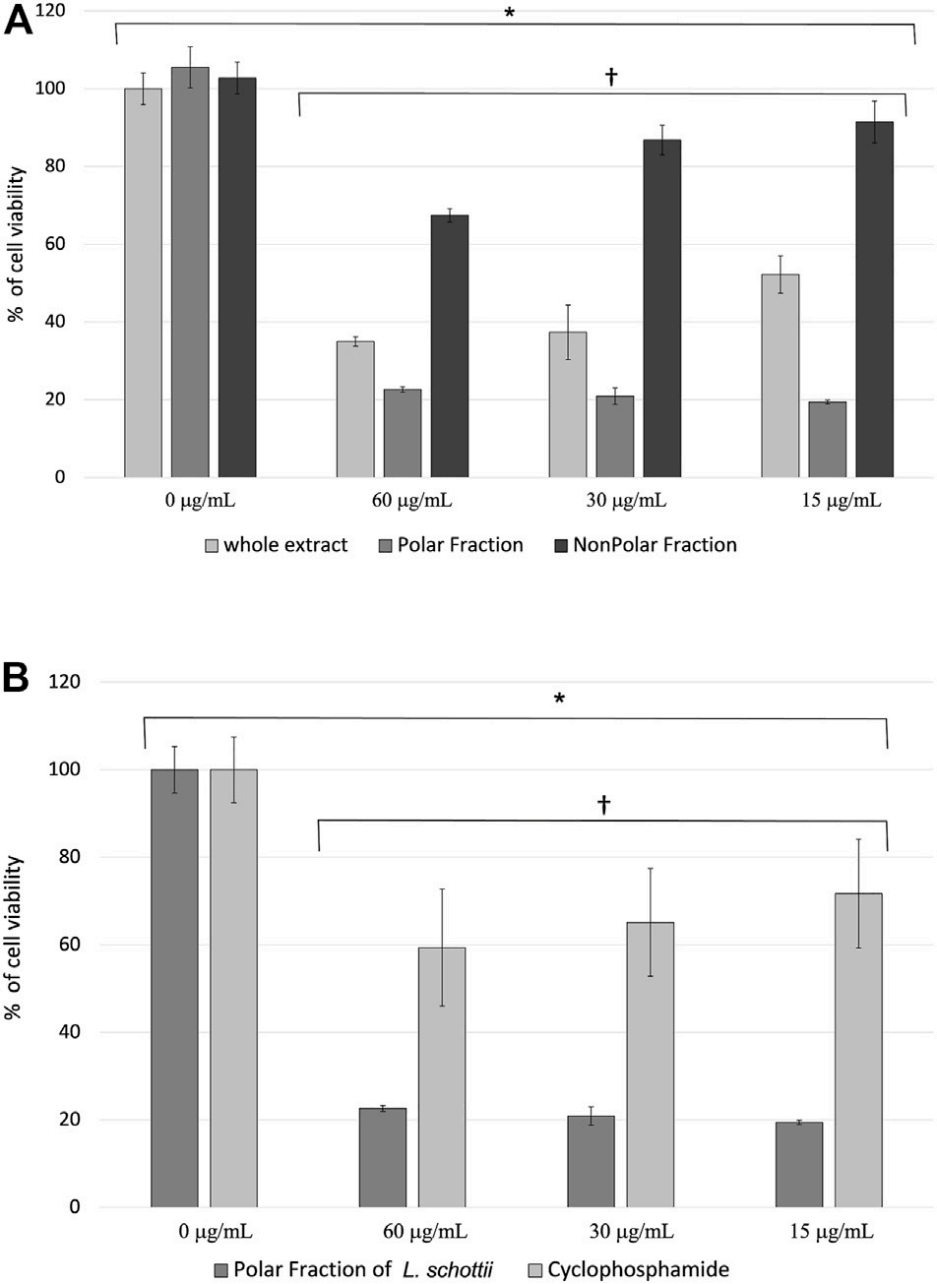
**(A)** Percentage of cell viability ± standard error of murine lymphoma L5178Y cells treated with the ethanolic extract and polar and nonpolar fractions of *Lophocereus schottii* (µg/mL). Values are the average of % cell viability. * p < 7.3 X 10^−8^. † p <1.7 X 10^−15^. **(B)** Percentage of cell viability ± standard error of murine lymphoma L5178Y cells treated with the polar fraction of the ethanolic extract of *Lophocereus schottii* (PFLs) and cyclophosphamide (µg/mL). Values are the average of the % of cell viability. * p < 4.4 x 10^−4^. † p < 5.8 X 10^−10^.

Both fractions and ethanol extract of *L. schottii* were dry at room temperature, then were weighed. The solids obtained of hexane and ethanolic extract were dissolved first with DMSO (Sigma-Aldrich Co., LLC, United States), and then diluted with culture medium RPMI 1640 (Sigma-Aldrich Co., LLC, United States), to obtain an initial concentration of 60 μg/mL, which contain DMSO at 1% (v/v). DMSO was not used to dissolve polar fraction. Consequently, the control experiments (indicated as 0 μg/mL in Figure 1A) for the whole extract and the nonpolar fraction contain equivalent concentrations of DMSO in the culture media. That was not the case for the polar fraction, in which no DMSO was needed. The initial concentration was serially diluted (1:2) for each sample. Before testing, all samples were sterilized using 0.22 µm pore syringe filters (Dualex, Merck Millipore Ltd, United States). The concentrations used to test samples were 60, 30, and 15 μg/mL. Each concentration was tested in triplicate and the experiment was repeated three times in different days.

### Qualitative Determinations of Phytochemicals in Different *L. schottii* Extracts and Fractions

Aliquots were taken from the ethanolic extract concentrates as well as the polar and nonpolar fractions of *L. schottii* to qualitatively determine the presence of alkaloids, steroids, flavonoids, terpenoids, phenols, and quinones, using the methods outlined in Table 1 (Jasnie, 2009; Jara-Beltrán, 2013).

**TABLE 1:** Methods for the qualitative identification of the phytochemicals of ethanolic extract and polar and nonpolar fractions of *Lophocereus schottii*.

Phytochemical compound	Identification method	Presence in the extract
Alkaloids	Mayer’s reagent	White precipitation
Steroids	Thin layer chromatography (TLC). Mobile phase: Petroleum ether/Ethyl ether/Ac. Acetic 80: 20: 1	Stains revealed with Liebermann-Burchard reagent
Flavonoids	Shinoda reaction	Color change to pink, red, purple, or orange
Terpenoids	Concentrated sulfuric acid	Color change to black
Phenols	5% Ferric chloride	Color change to black or blue
Quinones	5% potassium hydroxide	Color change to purple and red

### Identification of the Compounds From the Different Extracts by UPLC-MS

UPLC-MS analysis of the extracts was performed as follows. There was 1 mg of sample dissolved into 1 mL of HPLC grade solvent (for the whole extract and the polar fraction we used methanol, and for the nonpolar fraction we used 1:9 ethyl acetate:acetonitrile) and filtered through a 0.45 µm syringe filter for analysis. UPLC-MS analysis was carried out using an ACQUITY UPLC system liquid chromatography equipment coupled to a QDA^®^ mass detector from Waters (Milford, MA) ACQUITY UPLC CORTECS^®^ C18 column 1.6 µm 3.0 x 100 mm. Column temperature 40°C, autosampler temperature 15°C. Elution was achieved with 0.1% formic acid in water (phase A), acetonitrile (phase B), and 5 mM ammonium acetate (phase C). Flow rate was 0.3 mL/min, injection volume was 5 μL. The composition of the solvents over time was as follows: initial A: 5%; B: 85%; C: 10%, after 3.0 min increase A: 15%; B: 75%; C: 10%, changing at 10.0 min. A: 5%; B: 85%; C: 10%. Running time 15.0 min.

Data analysis was performed with MassHunter Qualitative Analysis B.06.00 (Agilent Technologies, Santa Clara, CA, United States). The characterization of compounds was carried out by the generation of candidate formula with a mass accuracy limit of 10 ppm. The tentative identification of the components was realized using open access web databases: HMDB, PhytoHub, and by comparison with the reported data in the literature.

### Lymphoma Cell Line

L5178Y cells originated in the 1950s when their first *ex vivo* predecessors were isolated as 3-methylcholanthrene-induced lymphocytic neoplasms from DBA/2J mice, named L5178 (McKinzie and Revollo, 2017), and were maintained in ascetic form by weekly intraperitoneal (i.p.) passages of 1 x 10^6^ cells in syngeneic BALB/c mice (H-2d/d) (Nugraha et al., 2019).

### Splenocytes From Balb/c Mice

Murine splenocytes were obtained as a primary culture by disintegrating the spleen in sterile Petri dishes with Hank’s balanced solution (Sigma-Aldrich Co., LLC, United States) and separated with Histopaque with a density of 1.083 g/mL (HISTOPAQUE 1083 Sigma-Aldrich Co., LLC) by centrifugation at 1500 rpm for 30 min at 20°C.

### Proliferation and Viability of L5178Y Cells and Splenocytes Assay

The cell proliferation effects of *L. schottii* extract and the polar and nonpolar fractions were evaluated *in vitro* using L5178Y cells and murine splenocytes, the latter either were or were not stimulated with 5 μg/mL of mitogen concanavalin A (ConA) (Sigma-Aldrich Co., LLC, United States). Cultures of 3 x 10^4^ and 2 x 10^5^ cells per well, respectively, were seeded in 96-well plates (CORNING 96 well microplates, United States) at a total volume of 200 µL. Cells were incubated in the presence or absence of various concentrations of *L. schottii* extracts and polar and nonpolar fractions (60, 30, and 15 μg/mL). Cells were cultured in RPMI 1640 medium (Sigma-Aldrich Co., LLC, United States), pH 7; supplemented with 10% fetal calf serum (Calf Serum, GIBCO BRL) and 1% antibiotic (solution containing 10,000 units/mL penicillin, 10 μg/mL streptomycin and 25 μg/mL Fungizone^®^ antifungal) (Thermo Fisher Scientific) with an incubation time of 24–72 h at 37°C under 5% CO_2_ and 95% humidity in a cell incubator model 2300 INCUBATOR (VWR Scientific Products, Radnor, PA, United States). An MTT [3-(4,5-Dimethyl-2-thiazolyl)-2,5-diphenyl-2H-tetrazolium bromide] (Sigma-Aldrich, St Louis, MO, United States) colorimetric assay was used to assess cell viability; optical density was measured at 595 nm with a Multiskan microplate reader model FC (Thermo Fisher Scientific, Finland). The viability percentage of the culture cells was estimated from the relative growth as follows: % Viability = (OD treated cells/OD not treated cells) x 100 (Navarro-Salcedo et al., 2017).

### Viability Assay of L5178Y Murine Lymphoma Cells Treated With the Polar Fraction of Ethanolic Extract of *L. schottii* (PFLs) and Cyclophosphamide

Viability of the L5178Y cells treated with the polar fraction of *L. schottii* (PFLs) ethanolic extract and cyclophosphamide (PISA Farmacéutica, Guadalajara, Mexico) was compared in the same culture conditions mentioned above. Cyclophosphamide (CTX), an alkylating agent, is a widely acknowledged anticancer chemotherapeutic agent used alone or in combination with other medicines as part of the mainstream therapy of several human malignancies (Liu et al., 2018; Hong et al., 2019).

### Determination of Effective Concentration 50 (EC_50_)

Effective concentration 50 (EC_50_) of PFLs was determined by the trypan blue exclusion assay. There was 15 µL of the culture taken and placed in an Eppendorf tube in which another 15 µL of 0.4% trypan blue (Sigma-Aldrich Co., LLC, United States) had previously been deposited, and 100 cells were counted by light microscope (Karl Zeiss, Germany) to calculate cell survival. Each experiment was repeated four times (Hong et al., 2019).

### Statistical Analysis

Cell proliferation assays were evaluated with the analysis of variance test (ANOVA) and Fisher’s least significant differences (LSD), with *p* < 0.05 considered to be significant. The values are represented as the percentage (%) of viable cells, considering that cells not treated with the *L. schottii* extracts (ethanolic extract and the polar and nonpolar fractions) correspond to 100%. The data represent the mean value of the percentage of viable cells ± the standard error (SE) of five triplicate independent experiments.

## Results

### Qualitative Determinations of Phytochemicals in Different Extracts and Fractions of *L. schottii*

Phytochemical generic compound presence or absence was determined from the ethanolic extract and from the polar and nonpolar fractions of *L. schottii*. Results are presented in Table 2.

**TABLE 2:** Phytochemical generic compounds present in the ethanolic extract and in polar and nonpolar fractions of *Lophocereus schottii*.

Phytochemical compound	Identification method	Complete ethanolic extract	Polar fraction	Nonpolar fraction
Alkaloids	Mayer’s reagent	+	+	+
Steroids	Thin layer chromatography (TLC)	+	−	+
Flavonoids	Shinoda reaction	+	+	+
Terpenoids	Concentrated sulfuric acid	+	+	−
Phenols	5% Ferric chloride	+	+	+
Quinones	5% potassium hydroxide	+	−	+

### Molecular Identification of Phytochemicals in Different Extracts and Fractions of *L. schottii*

Based on the qualitative determination results, we decided to perform the tentative identification, by UPLC-MS, of the secondary metabolites present in the extracts. We focused on alkaloids, flavonoids, terpenoids, and sterols. We identified 21 compounds present in the ethanolic extract. In addition, we identified 20 and 28 compounds in their polar and nonpolar fractions, respectively. Those compounds correspond mainly to alkaloids and flavonoids, but terpenoids and sterols were also present (Table 3), as well as some unknown compounds.

**TABLE 3:** Phytochemical compounds present in the ethanolic extract and polar and nonpolar fractions of *Lophocereus schottii*. The percentages were obtained by summation of those obtained for each extract or fraction.

Phytochemical compound	Complete ethanolic extract (%)	Polar fraction (%)	Nonpolar fraction (%)
Alkaloids	13.56	97.15	11.88
Steroids	2.80	−	55.16
Flavonoids	80.70	0.23	4.43
Terpenoids	0.25	0.07	0.36
Unknown	2.70	2.48	28.14

The complete list of tentatively identified secondary metabolites from the ethanol extract is presented in Table 4. The identified secondary metabolites from the polar extract and nonpolar fraction are presented in Table 5 and Table 6, respectively.

**TABLE 4:** Secondary metabolites present in the ethanolic extract of *Lophocereus schottii*.

Peak	RT (min)	% Area	Exp m/z [M + H]^+^	Molecular	Tentative compound	Metabolite class	Ref.
				Formula	Identification		
1	0.86	0.1629	576.43	C_35_H_60_O_6_	Sitosterol glucoside	Sterol	Münger et al. (2018)
2	0.91	0.4053	595.17	C_27_H_30_O_15_	Kaempferol-3-neohesperidoside	Flavonoid	Joo et al. (2020)
3	1.09	2.5	411.36	C_29_H_46_O	Poriferasta-8,22,25-trienol	Sterol	Münger et al. (2018)
4	1.1	0.7904	398.10	C_20_H_19_N_3_O_6_	Tryptophan-betaxanthin	Alkaloid	Polturak et al. (2018)
5	1.13	0.2144	315.08	C_17_H_14_O_6_	2′,7-Dihydroxy-4′,5’-	Flavonoid	Tang et al. (2019)
					dimethoxyisoflavone		
6	1.16	11.782	250.17	C_15_H_23_NO_2_	Lophocerine	Alkaloid	Kim et al. (2020)
7	1.18	0.7723	238.25	C_13_H_19_NO_3_	Anhalotine	Alkaloid	Unger et al. (1980)
8	1.23	78.935	287.06	C_15_H_11_O_6_	Kaempferol	Flavonoid	Joo et al. (2020)
9	1.24	−	250.21	C_14_H_19_NO_3_	Peyophorine	Alkaloid	Kapadia et al. (1968)
10	1.26	0.2674	319.04	C_15_H_10_O_8_	Myricetin	Flavonoid	Tang et al. (2019)
11	2.38	1.5528	440.35	−	Unknown	−	−
12	2.42	0.1109	395.81	−	Unknown	−	−
13	2.43	0.4112	291.23	C_15_H_14_O_6_	Epicatechin	Flavonoid	Mittal et al. (2013)
14	2.52	0.1692	306.37	C_16_H_19_NO_5_	Peyonine	Alkaloid	Kapadia et al. (1968)
15	4.83	0.2533	448.09	C_21_H_20_O_11_	Quercetin rhamnoside	Flavonoid	Jang et al. (2018)
16	4.94	0.2066	434.81	C_21_H_22_O_10_	Quercetin xyloside	Flavonoid	Jang et al. (2018)
17	8.28	0.1337	591.42	C_39_H_58_O_4_	Schottenol ferulate	Sterol	Djerassi et al. (1958)
18	10.42	0.531	503.81	−	Unknown	−	−
19	12.1	0.1927	279.23	−	Unknown	−	−
20	13.66	0.2488	543.48	C_40_H_62_	Phytofluene	Terpenoid	Amaya-Cruz et al. (2019)
21	14.42	0.0504	208.17	C_12_H_17_NO_2_	Salsolidine	Alkaloid	Unger et al. (1980)
22	1.23	0.3095	286.82	−	Unknown	−	−

**TABLE 5:** Secondary metabolites present in the polar fraction of the extract of *Lophocereus schottii.*

Peak	RT (min)	% Area	Exp m/z [M + H]^+^	Molecular formula	Tentative compound	Metabolite class	Ref.
					Identification		
1	1.2	85.636	250.17	C_15_H_23_NO_2_	Lophocerine	Alkaloid	Kim et al. (2020)
2	1.21	10.68	250.21	C_14_H_19_NO_3_	Peyophorine	Alkaloid	Kapadia et al. (1968)
3	2.24	0.0262	418.25	C_20_H_19_O_10_	Kaempferol xiloside	Flavonoid	Jang et al. (2018)
4	2.46	0.3397	335.28	−	Unknown	−	−
5	2.53	1.1661	440.8	−	Unknown	−	−
7	3.61	0.0542	543.92	C_40_H_62_	Phytofluene	Terpenoid	Giacomoni et al. (2017)
8	5.33	0.1421	449.19	C_21_H_20_O_11_	Kaempferol 3-O-glucoside	Flavonoid	Jang et al. (2018)
9	6.33	0.0163	425.34	C_29_H_44_O_2_	Alpha-Tocotrienol	Terpenoid	Giacomoni et al. (2017)
10	7.33	0.0468	108.3	−	Unknown	−	−
11	9.04	0.1072	227.2	−	−	−	−
12	9.6	0.0905	279.58	−	Unknown	−	−
13	10.16	0.0217	450.41	C_21_H_21_O_11_	Cyanidin 3-O-glucoside	Flavonoid	Giacomoni et al. (2017)
14	10.29	0.0349	581.15	C_26_H_28_O_15_	Kaempferol 3-O-xylosyl-glucoside	Flavonoid	Giacomoni et al. (2017)
15	12.33	0.7682	359.33	C_18_H_18_N_2_O_6_	Phenylalanine-betaxanthin	Alkaloid	Giacomoni et al. (2017)
16	12.65	0.0765	281.78	C_18_H_34_O_2_	Coumaroyl malic acid	Phenolic acids	Giacomoni et al. (2017)
17	12.83	0.0615	461.4	C_22_H_26_N_2_O_9_	2,17-didecarboxy-neobetanin	Alkaloid	Giacomoni et al. (2017)
18	13.29	0.2238	502.19	−	Unknown	−	−
19	13.68	0.0589	476.21	−	Unknown	−	−
20	2.6	0.4045	570.24	−	Unknown	−	−
21	7	0.0433	573.03	−	Unknown	−	−

**TABLE 6:** Secondary metabolites present in the nonpolar fraction of the extract of *Lophocereus schottii.*

Peak	RT (min)	% Area	Exp m/z [M + H]+	Molecular formula	Tentative compound identification	Metabolite class	Ref.
1	1.16	3.8769	266.22	−	Unknown	−	−
2	1.17	7.3774	476.21	−	Unknown	−	−
3	1.24	1.2883	112.08	C_5_H_9_N_3_	Histamine	Alkaloid	Giacomoni et al. (2017)
4	1.26	0.7183	294.21	C_15_H_19_NO_5_	Mescaline-succinimide	Alkaloid	Duke (1992)
5	1.41	0.6163	307.27	C_15_H_14_O_7_	Gallocatechin	Flavonoid	Giacomoni et al. (2017)
6	1.43	3.9839	250.17	C_15_H_23_NO_2_	Lophocerine	Alkaloid	Kim et al. (2020)
7	1.48	10.3683	415.18	C_29_H_50_O	Beta sitosterol	Sterol	Giacomoni et al. (2017)
8	1.49	3.1475	288.23	C_15_H_11_O_6_	Cyanidin	Flavonoid	Giacomoni et al. (2017)
9	1.85	1.4954	316.31	−	Unknown	−	−
10	2.32	5.1758	415.39	C_29_H_50_O	Scottenol	Sterol	Zakharenko et al. (2020)
11	2.35	8.3139	528.25	−	Unknown	−	−
12	2.64	6.7004	387.36	C_27_H_46_O	Cholesterol	Sterol	Münger et al. (2018)
13	2.67	3.4124	526.24	−	Unknown	−	−
14	2.91	32.9107	397.81	C_28_H_44_O	Ergosterol	Sterol	Giacomoni et al. (2017)
15	3.13	0.3168	533.05	−	Unknown	−	−
16	6.17	0.6625	299.08	C_17_H_14_O_5_	Apigenin 7,4′-dimethyl ether	Flavonoid	Giacomoni et al. (2017)
17	6.99	0.3108	499.45	−	Unknown	−	−
18	7.27	1.6881	101.49	−	Unknown	−	−
19	8.06	0.7769	238.19	C_13_H_19_NO_3_	Anhalotine	Alkaloid	Unger et al. (1980)
20	8.18	0.5746	348.13	C_15_H_16_N_4_O_6_	Histidine-betaxanthin	Alkaloid	Amaya-Cruz et al. (2019)
21	8.75	0.1739	113.98	−	Unknown	−	−
22	9.17	0.3561	279.82	−	Unknown	−	−
23	9.38	0.3576	599.79	C_40_H_56_O_4_	Violaxanthin	Terpenoid	Amaya-Cruz et al. (2019)
24	9.68	0.2978	114.15	−	Unknown	−	−
25	10.07	0.5244	106.25	−	Unknown	−	−
26	11.70	0.1590	122.95	C_8_H_11_N	Phenethylamine	Alkaloid	Giacomoni et al. (2017)
27	13.58	0.3743	208.61	C_12_H_17_NO_2_	Salsolidine	Alkaloid	Unger et al. (1980)
28	14.82	4.0402	387.12	C_18_H_16_N_2_O_8_	Betanidin	Alkaloid	Giacomoni et al. (2017)

From the analysis of the identified secondary metabolites, we observed that only lophocerine was present in all three extracts, being a characteristic compound of *L. schottii*. In addition, this compound was among the more abundant in the extracts, mainly in the polar fraction, composing almost 86%. It is also the second most abundant in the total extract, composing almost 12%, and is present in the nonpolar fraction, but only at 4%.

Two compounds are present both in the ethanolic extract and in the nonpolar fraction: salsolidine and anhalotine. Greater similarity exists between the ethanolic and polar fraction, sharing as they do peyophorine, phytofluene, and modified forms of kaempferol. As expected, between the polar and nonpolar fractions there were no common compounds, except for lophocerine.

Given the anticancerogenic activity that the ethanolic extract and polar and nonpolar fractions present, we expected to identify compounds that were previously reported with anticancer activity. As previously mentioned, the most significant compound present was lophocerine. The presence of several flavonoids in the extract and two fractions was noteworthy, the most interesting being the seven compounds present in the ethanolic extract: kaempferol and kaempferol-3-neo-hesperidoside, epicatechin, myricetin, and quercetin (-xyloside and -rhamnoside) and 2′,7-Dihydroxy-4′,5′-dimethoxyisoflavone. This represents almost 81% of the extract, with kaempferol being the most abundant (79%). Also of note were the four compounds present in the polar fraction: three forms of kaempferol (3-O-glucoside, xyloside, and 3-O-xylosyl-glucoside), and cyanidin 3-O-glucoside. However, all four only represent 0.22% of the extract. Finally, in the nonpolar fraction there were also present three flavonoids with reported anticancer activity: gallocatechin, cyanidin, and apigenin (although in this case in the form of apigenin 7,4′-dimethyl ether). They represented only 4.4% of the extract, equivalent to the lophocerine present.

Regarding alkaloids, although we found several compounds in the three extracts, with lophocerine being the most abundant, only three had previously been reported with anticancerogenic activity, one case for each. Those were: tryptophan-betaxanthin in the ethanolic extract (0.79%), phenylalanine-betaxanthin in the polar fraction (0.77%), and phenethylamine in the nonpolar fraction (0.16%). Of additional interest, we also found peyonine and peyophorine, the latter in common between ethanol extract and polar fraction, and the second being most abundant here, with 10.7%.

Regarding terpenoids, only one was present in the ethanolic extract: phytofluene (0.25%), which was also present in the polar fraction (0.05%), in addition to alpha-tocotrienol (0.02%). Finally, only one terpenoid was present in the nonpolar fraction: violaxanthin (0.36%). Concerning sterols with reported anticancerogenic activity, we found sitosterol glucoside in the ethanolic extract (0.16%) and ergosterol in the nonpolar fraction (32.91%).

Finally, from the 63 identified different compounds (69 minus the 6 that were repeated once or twice among the extracts), a total of 23 compounds were unknown, most of them (12) in the nonpolar fraction, four in the ethanol extract and seven in the polar fraction. This means that about one-third of the compounds present in the extracts are still unidentified. We now at least know their molecular weights and retention times by UPLC-MS to begin their future study.

### Determination of Percentage of Viability of Ethanolic Extract and Fractions of *L. schottii*

The extract and the fractions of *L. schottii* show decreased viability in L5178Y cells (*p* < 7.3 x 10^−8^), and the effect between concentrations is also significant (*p* < 1.7 x 10^−15^). There is a dose-dependent effect in the concentrations used of both the ethanolic extract and the nonpolar fraction, but not in the polar fraction (Figure 1A). The polar fraction was found to produce the lowest viability percentage in L5178Y lymphoblast, on average two- and fourfold that of the ethanolic extract and the nonpolar fraction, respectively (Table 7).

**TABLE 7:** Average of the percent viability of murine lymphoma L5178Y cells treated with the ethanolic extract and polar and nonpolar fractions of *Lophocereus schottii* (µg/mL). *p* < 1.7X10-15.

Concentration	Whole extract (%)	Polar fraction (%)	Nonpolar fraction (%)
60 μg/mL	35	23	67
30 μg/mL	37	21	87
15 μg/mL	52	19	91
Average % of cell viability	42	21	82

### Effect of the Polar Fraction of the Ethanolic Extract of *L. schottii* and of Cyclophosphamide on the Viability of L5178Y Murine Lymphoma Cells

The polar fraction of *L. schottii* (PFLs) inhibits the viability of L5178Y cells three times as much as cyclophosphamide (*p* < 5.8 x 10^−10^). Both extract and polar fraction significantly inhibit the proliferation of treated murine lymphoblasts (p < 4 x 10^−4^). The three concentrations of cyclophosphamide and polar fraction are the same (Figure 1B).

### Effect of the Polar Fraction of *L. schottii* on the Viability of Healthy Murine Splenocytes

**FIGURE 2:**
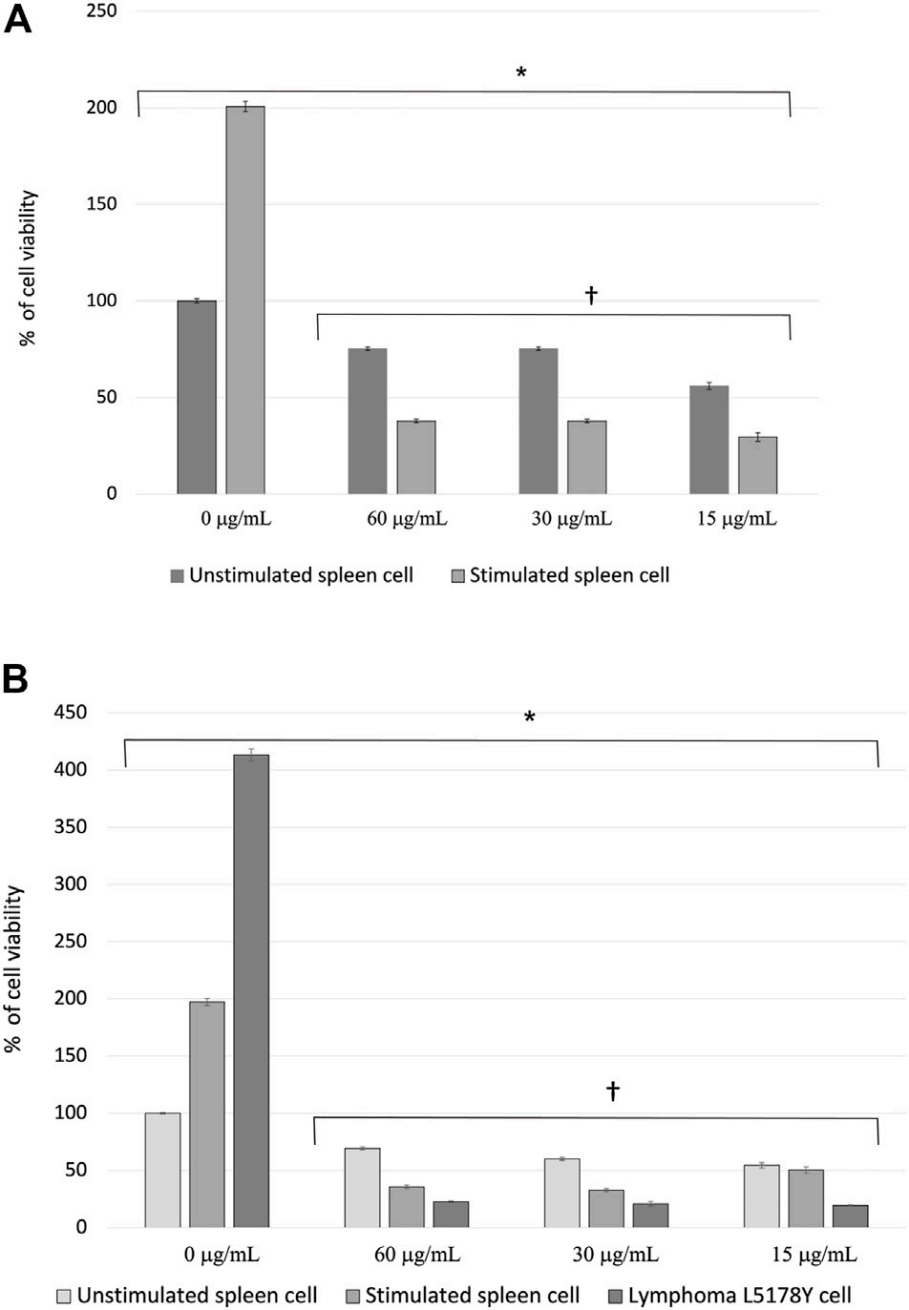
**(A)** Percentage of cell viability ± standard error of splenocytes of healthy mice stimulated and not stimulated with the mitogen Concanavalin A (ConA) treated with the polar fraction of the ethanolic extract of *Lophocereus schottii* (µg/mL). Values are the average of % cell viability. *p < 4 X 10^−4^ † p < 2.6 X 10^−13^. **(B)** Percentage of cell viability ± standard error of splenocytes from healthy mice stimulated and not stimulated with the mitogen Concanavalin A (ConA) and murine lymphoma L5178Y cells treated with the polar fraction of the ethanolic extract of *Lophocereus schottii* (µg/mL). *p < 8.9 X 10^−11^. † *p* < 0.05.

The effect of the PFLs on the viability of healthy cells was determined by assessing the cultures of splenocytes at rest and stimulated with the mitogen ConA. In untreated spleen cells, the viability of splenocytes stimulated with ConA was twice that of nonstimulated cells. The difference between cells treated with the PFLs and those not treated was significant (p < 4 x 10^−4^) regardless of the ConA stimulus. Furthermore, the effect of the PFLs was significantly greater in the inhibition of cell viability (*p* < 2.6 x 10^−13^) in stimulated cells than in those not stimulated with ConA (Figure 2A).

Figure 2B shows the viability percentage of the nonstimulated and stimulated splenocytes and L5178Y cells under the effect of the concentrations of the PFLs. There is 100% cell viability presented by the group of splenocytes neither stimulated with the mitogen ConA nor treated with the PFLs, and the viability percentages of the splenocytes stimulated with ConA and the L5178Y cells not treated with the PFLs are two- and fourfold higher, respectively. On the other hand, the three concentrations of the PFLs used in each cell type are three times more effective in lymphoma cells than in healthy unstimulated splenocytes and twice as effective as splenocytes stimulated with ConA.

### Determination of Effective Concentration 50

**FIGURE 3:**
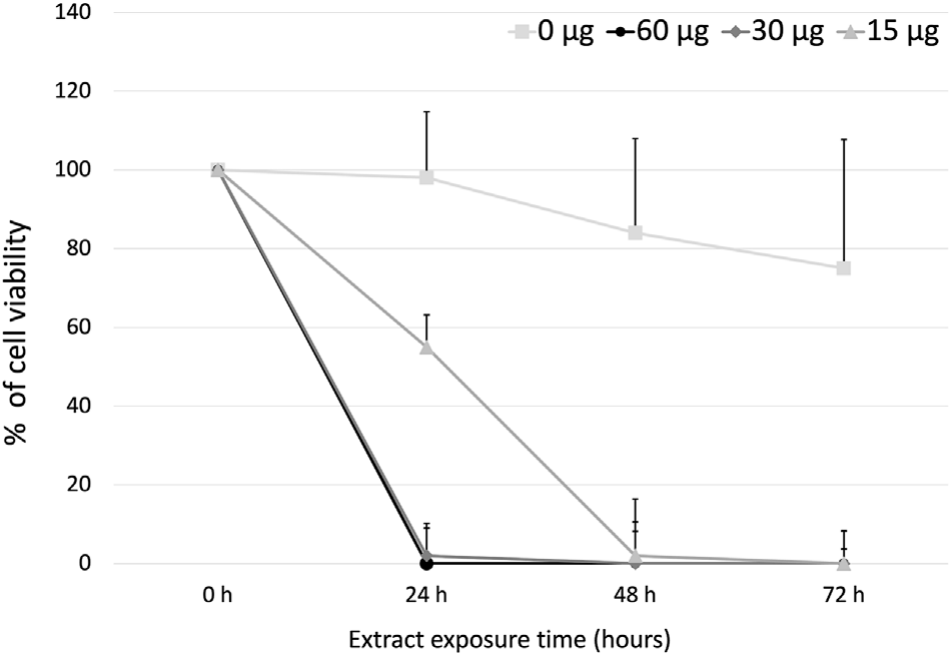
Effective concentration 50 (µg/mL) of the polar fraction of the ethanolic extract of *Lophocereus schottii* in murine lymphoma L5178Y cells. μg/mL.

The effective concentration 50 (EC_50_) of the PFLs on the cells of murine lymphoma L5178Y, was determined with the trypan blue exclusion assay. At 24 h, doses of 60, 30, and 15 μg/mL achieved 100, 98, and 55% inhibition, respectively. At 48 h, 15 μg/mL of the PFLs completely inhibited the L5178Y cells. At 72 h, no viable cells were found. The EC_50_ of the polar fraction of *L. schottii* was deduced to be 15 μg/mL at 24 h (Figure 3).

## Discussion

In this study, we found that the ethanolic extract of *L. schottii*, as well as its polar fraction, exhibit a remarkable antiproliferative activity, with higher activity in the latter. With the three doses used (60, 30, and 15 μg/mL) PFLs inhibited the cell viability of the ConA stimulated splenocytes and in L5178Y cells two and threefold more than in unstimulated cases (Figure 2B). The results show that among the phytochemicals most abundant in the total extract and in the polar and nonpolar fractions from *L. schottii*, there are many that had exhibited anticancerogenic activities. The most abundant was kaempferol (79%) in the total extract, and ergosterol (33%) in the nonpolar fraction. However, the fraction with the highest activity was the polar fraction, which is enriched in lophocerine (86%), and peyophorine (11%), two alkaloids without previously described anticancerogenic activity. Both are also present in the total extract, and consequently constitute new candidates as anticancerogenic phytochemicals as we proposed here.

The efficacy of the PFLs was compared with that of another chemotherapeutic agent for clinical use, cyclophosphamide. This is known to have an alkylating effect on DNA, forming cross-links between guanosine and cytidine, either between chains or interchains, with the cells with the highest proliferation being the most sensitive (Ahlmann and Hempel, 2016). In Figure 1B it is observed that the effect of the PFLs on the inhibition of the viability of the L5178Y cells is threefold greater in each of the three concentrations used compared with cyclophosphamide. This difference could be due to the form of action of the lophocerine (National Center for Biotechnology Information, 2021), the more abundant alkaloid found in *L. schottii* (Djerassi et al., 1958; O'Donovan and Horan, 1968; Bobbitt and Chou, 1959), which belongs to a class of isoquinoline, and has anticancer effects. One isoquinoline alkaloid, tetrahydroisoquinoline (Dembitsky et al., 2015), acts on different cellular metabolism pathways including: the inhibition of topoisomerases (Nguyen et al., 2015), the arrest of the G0/G1 phase of the cell cycle, the activation of caspases (Sun et al., 2018), apoptosis (Ding et al., 2016), the disruption of mitochondrial membrane potential (Iqbal et al., 2017), the activation of the p53 pathway (Liu et al., 2019), and the inhibition of the enzyme cyclooxygenase-2 (Hashmi et al., 2018). Most of these metabolic pathways occur in stages of the cell cycle like the DNA synthesis phase, in mitosis or in G1.

In addition, other alkaloids are known to have anticancer and immunostimulatory properties (Kalmarzi et al., 2019; Nugraha et al., 2019). Some, such as those of the vinca subset (vincristine and vinblastine), isolated from *Catharanthus roseus* (*C. roseus* Apocynaceae), bind to specific sites called tubulin heterodimers which interrupt the functions of microtubules and stop the cell cycle at metaphase, preventing their polymerization and avoiding the formation of the achromatic spindle (Kumar et al., 2017; Naaz et al., 2019; Borys et al., 2020). Consequently, lophocerine can act over cell cycle regulation mechanisms, probably acting preferentially on cells with exacerbated mitotic activity, as other alkaloids do, altering some of the metabolic pathways of the cell cycle, such as the inhibition of microtubule polymerization (Borys et al., 2020), cyclin activity (Ding et al., 2016; Sun et al., 2018) or activation of the p53 pathway (Liu et al., 2019). However, there is still no evidence documenting the precise mechanism of action of lophocerine, so further studies must be undertaken to document the mitogenic pathways regulated by this alkaloid.

Besides alkaloids, we cannot rule out the participation of flavonoids, terpenoids, and even sterols present in *L. schottii* extracts which could contribute to their anti-cancerinogenic properties. Several flavonoids are known for their antioxidative properties and for their anti-cancer activity. Kaempferol, the most abundant flavonoid in all three extracts, has antineoplastic effects with mechanisms that have recently been elucidated. *In vitro*, kaempferol reduces neoplastic cell proliferation through cyclin inhibition, promotes caspase activation and other proapoptotic proteins, induces DNA condensation through histone phosphorylation (Zhu and Xue, 2019), induces autophagic mechanisms and programmed cell death (Lee et al., 2017), and inhibits phenotypes associated with invasiveness and metastasis (Kim et al., 2018). Similar effects have been reported for myricetin (Jiang et al., 2019), epicatechin (Ijaz et al., 2018), and quercetin (Tang et al., 2020). However, their low bioavailability and lack of evidence from *in vivo* models (Li et al., 2020) present some difficulties for their application as potential therapeutic agents.

The presence of terpenoids in the three extracts is limited, with less than 0.5% in each. The two compounds of this class identified are phytofluene and alpha-tocotrienol. Some antitumor effects reported for these molecules include antiproliferation, programmed cell death induction (Yano et al., 2013), DNA fragmentation, and regulation of cell viability through hormone receptors modulation in prostate (Linnewiel-Hermoni et al., 2015) and breast (Hirsch et al., 2007) cancer cell lines.

We only found sterols in the ethanolic extract and the nonpolar fraction. Regarding sitosterol glucoside, there is evidence of antiproliferative activity in hepatic cells (Huh7 and HepG2) by activation of caspases 3 and 9 (Li et al., 2020). Ergosterol has an antitumor effect through multiple pathways including up-regulation of tumor suppressor genes (Li et al., 2015), induction of caspases (Han et al., 2019), attenuation of angiogenesis, and promotion of cell cycle arrest through inhibition of oncogenes like β-catenin, AKT, and c-MYC (Tan et al., 2017), among others.

However, since the relative number of flavonoids, terpenols, and sterols are exceedingly small in the PFL and ethanolic extract in comparison with alkaloids, we therefore infer that the antiproliferative effects observed in the L5178Y cells and stimulated spleen cells could be attributed to the more abundant compounds such as lophocerine.

Finally, it is important to note that the National Cancer Institute (NCI) of the United States of America considers plant extracts and pure compounds with values of inhibition of cell viability or cytotoxicity with ED_50_ ≤ 30 μg/mL and ≤4 μg/mL, respectively, as active anticancer substances (Suffness et al., 1991). Within these parameters, the PFLs would be considered as an anticancer substance, since the EC_50_ obtained at 24 h was 15 μg/mL with 55% cell inhibition and with 30 μg/mL eliminating the total number of L5178Y cells (Figure 3).

The limitations of this study present several possibilities. The mechanism of action of the combination of the diverse compounds from *L. schottii* is still unknown. There is no evidence documenting the precise mechanism of action of lophocerine; however, the present results suggest the necessity for further research to test the identified phytochemical compounds independently and/or in diverse combinations in different human cancer cell lines through a phytochemical directed study, to isolate and characterize the chemicals responsible for the antiproliferative activity and to determine its mechanism of action. Verification of the unrecognized compounds in the different extracts is also necessary. Subsequent studies should also be aimed at establishing which metabolic pathways are affecting them, especially those involved in the cell cycle activation processes and apoptosis inhibition, and the possible involvement of the alkaloid, lophocerine, in the regulation of mitogenic pathways.

## Conclusion

The present study clearly establishes that the complete ethanolic extract, and the polar and nonpolar fractions of *L. schottii* have a cytotoxic effect on L5178Y cells and on the splenocytes of healthy mice stimulated with the ConA mitogen. Additionally, the polar fraction has a significantly greater effect on the inhibition of cell viability in cells with exacerbated mitosis, respectively, three- and twofold greater in L5178Y cells and stimulated splenocytes than in the cells at rest. Likewise, it was determined that the PFLs are three times more effective than cyclophosphamide in inhibiting the viability of L5178Y cells. Finally, PFLs can be considered an anticancer substance since its EC_50_ of 15 μg/mL is within the parameters established by the NCI (≤30 μg/mL in a crude extract is considered an anticancer substance).

## Data Availability

The original contributions presented in the study are included in the article/Supplementary Material, further inquiries can be directed to the corresponding authors.
