# Supplementary material for: In vitro Anticancer Activity of the Polar Fraction From the Lophocereus schottii Ethanolic Extract
Source: Front Pharmacol. 2022 Apr 4;13:820381. doi: 10.3389/fphar.2022.820381 (PMC9014087; doi:10.3389/fphar.2022.820381)
Supplement: Supplementary file 1 [file DataSheet1.pdf]

Supplementary Figure. UPLC Chromatograms

In vitro anticancer activity of the polar fraction from the *Lophocereus schottii* ethanolic extract

Arturo Orozco-Barocio [1]*, Blanca Susana Robles-Rodríguez [1], María del Rayo Camacho-Corona [2], Luis Fernando Méndez-López [3], Marisol J. Godínez-Rubí [4], Jorge Peregrina-Sandoval [1], Gildardo Rivera [5], Argelia E. Rojas-Mayorquín [6], Daniel Ortuño-Sahagún [7]*.

a) Ethanolic extract

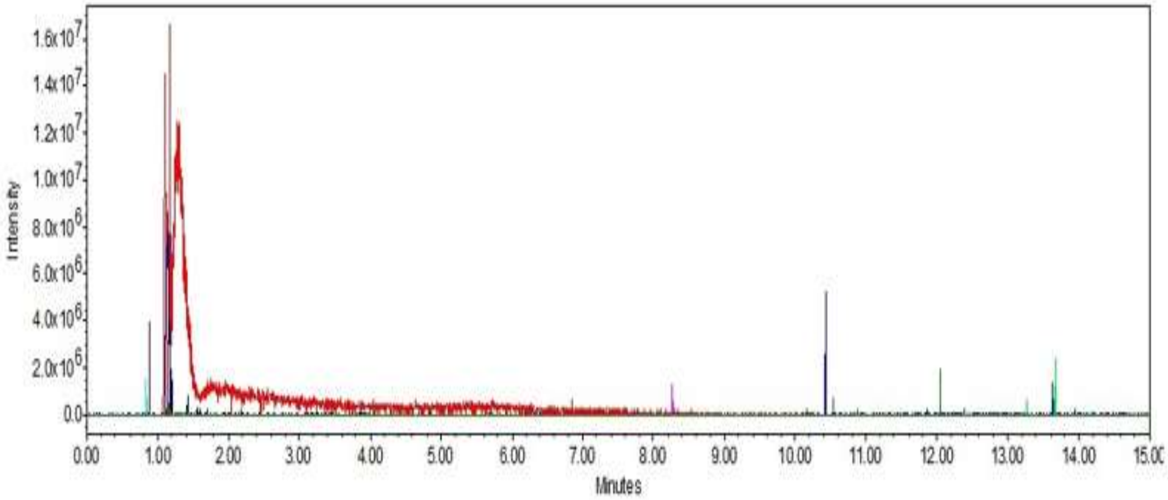

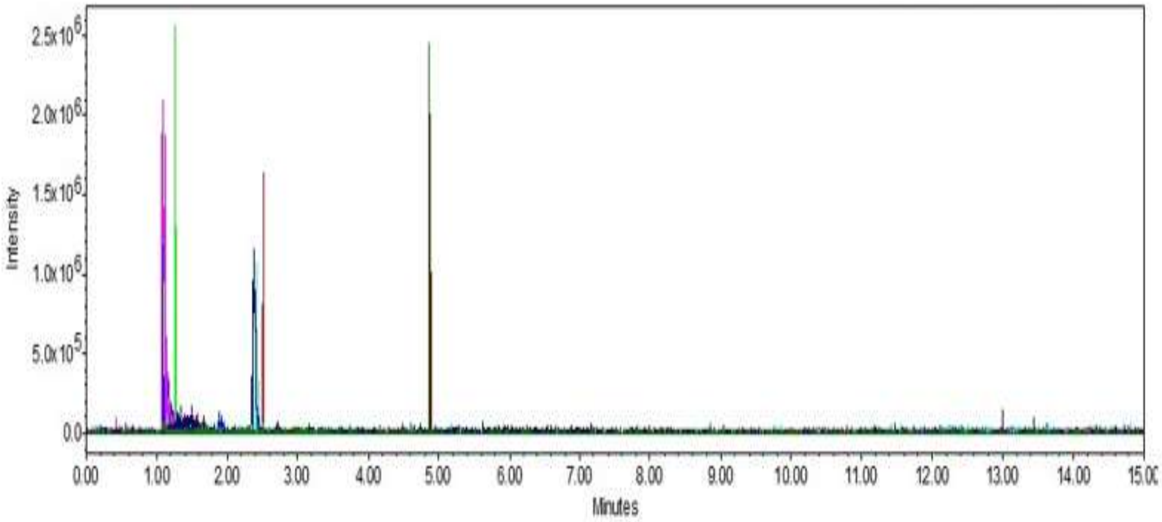

Supplementary Figure. UPLC Chromatograms

In vitro anticancer activity of the polar fraction from the *Lophocereus schottii* ethanolic extract

Arturo Orozco-Barocio [1]*, Blanca Susana Robles-Rodríguez [1], María del Rayo Camacho-Corona [2], Luis Fernando Méndez-López [3], Marisol J. Godínez-Rubí [4], Jorge Peregrina-Sandoval [1], Gildardo Rivera [5], Argelia E. Rojas-Mayorquín [6], Daniel Ortuño-Sahagún [7]*.

b) Ethanolic fraction (Polar fraction)

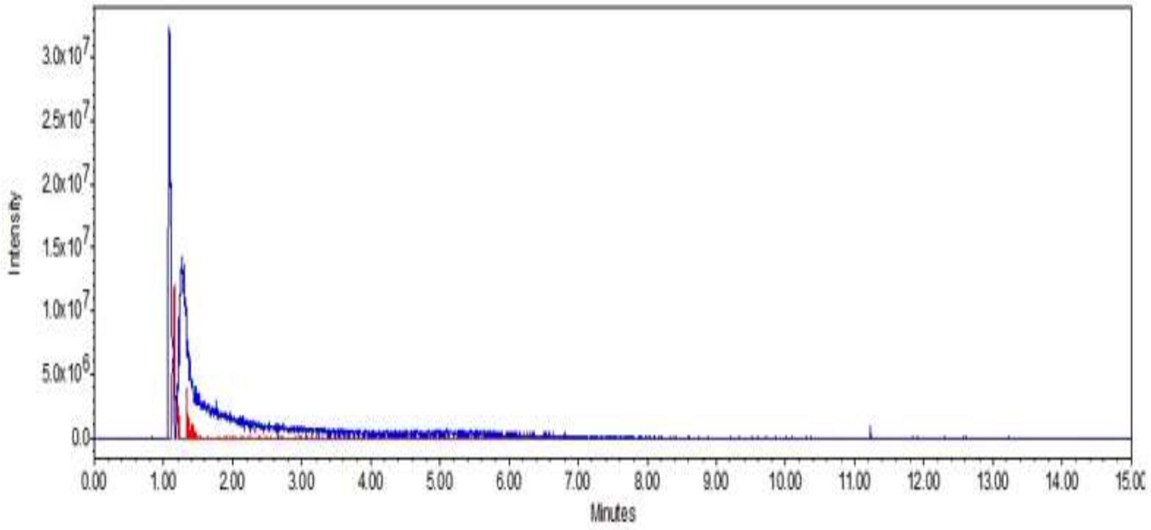

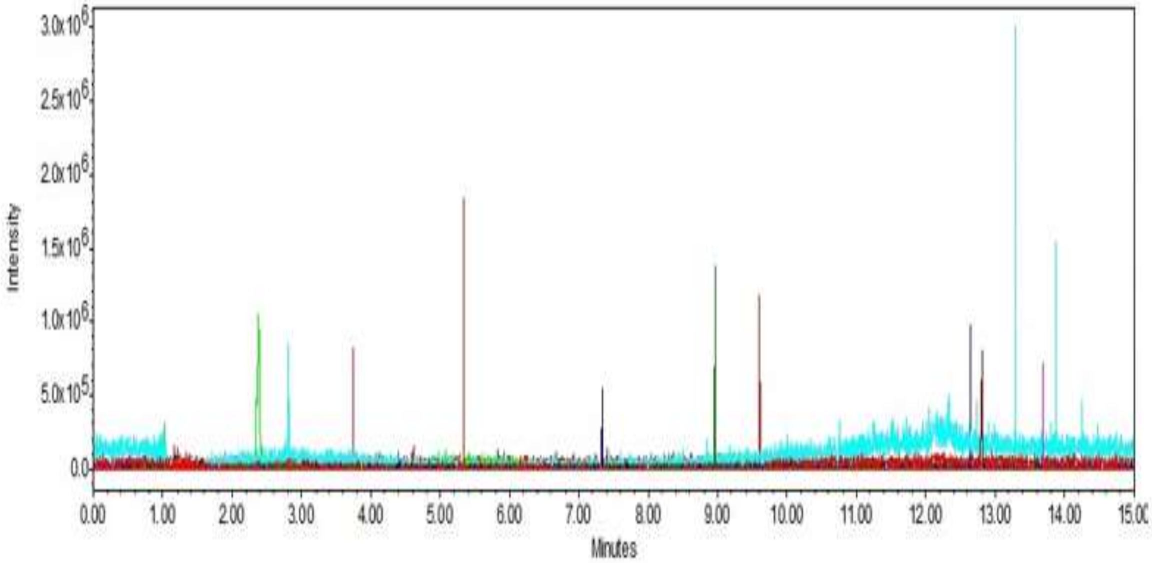

Supplementary Figure. UPLC Chromatograms

In vitro anticancer activity of the polar fraction from the *Lophocereus schottii* ethanolic extract

Arturo Orozco-Barocio [1]*, Blanca Susana Robles-Rodríguez [1], María del Rayo Camacho-Corona [2], Luis Fernando Méndez-López [3], Marisol J. Godínez-Rubí [4], Jorge Peregrina-Sandoval [1], Gildardo Rivera [5], Argelia E. Rojas-Mayorquín [6], Daniel Ortuño-Sahagún [7]*.

c) Hexanic fraction

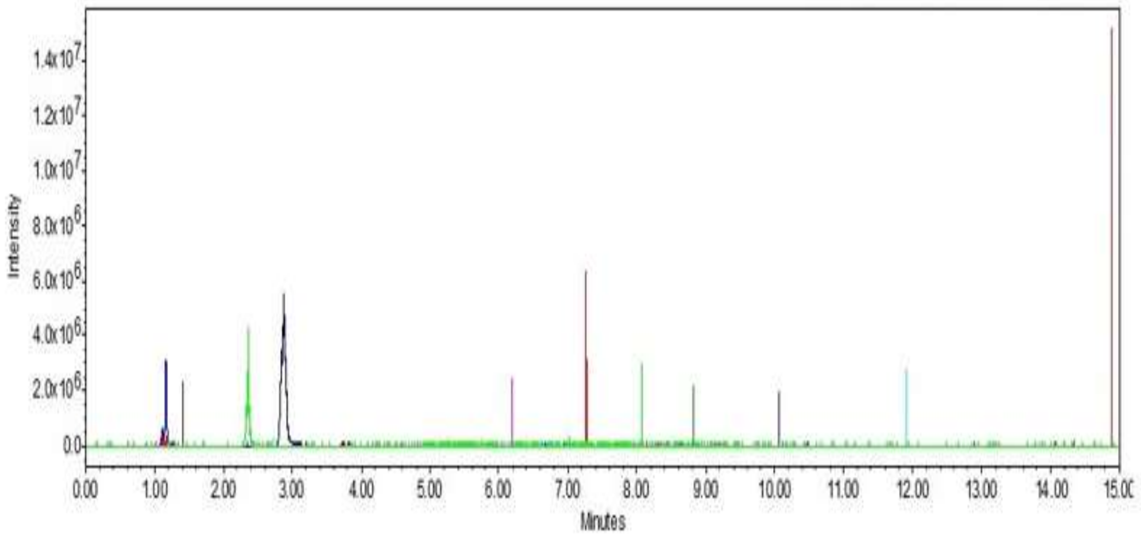

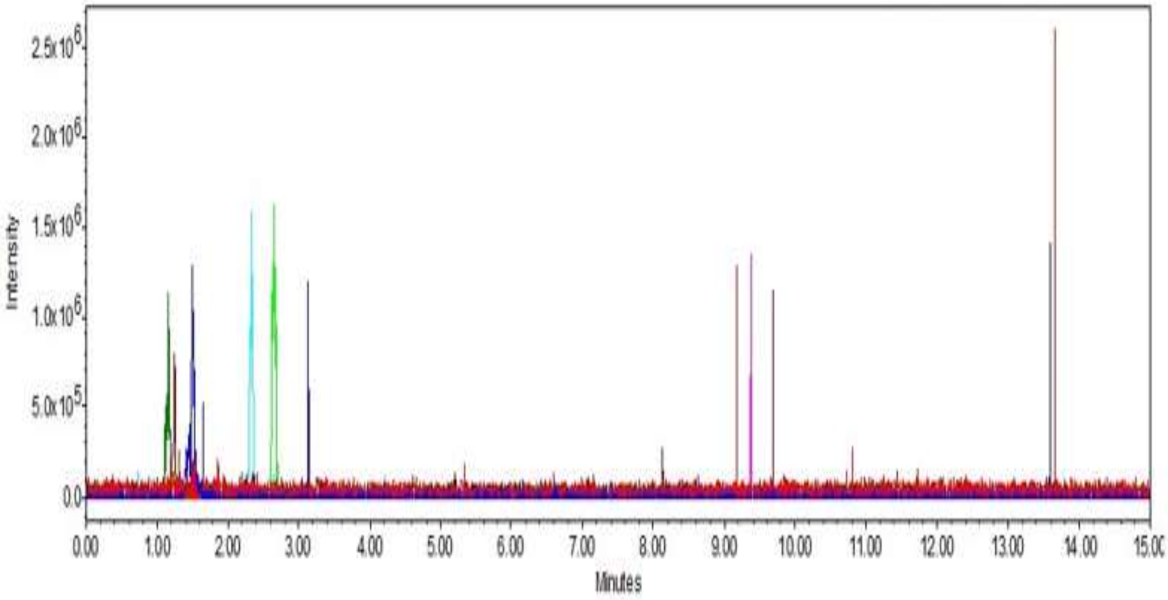

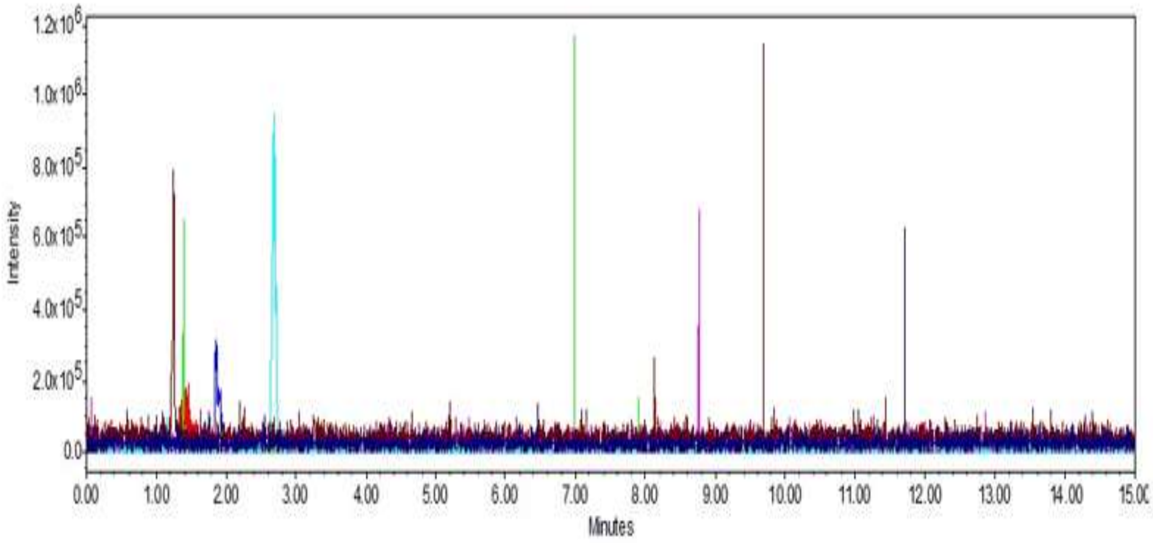
Intensity
Minutes
1.2x10^6
1.0x10^6
8.0x10^5
6.0x10^5
4.0x10^5
2.0x10^5
0.0
0.00 1.00 2.00 3.00 4.00 5.00 6.00 7.00 8.00 9.00 10.00 11.00 12.00 13.00 14.00 15.00
